# Effectiveness and Safety of Simultaneous Integrated Boost-Proton Beam Therapy for Localized Pancreatic Cancer

**DOI:** 10.1177/1533033818783879

**Published:** 2018-07-01

**Authors:** Tae Hyun Kim, Woo Jin Lee, Sang Myung Woo, Hyunjung Kim, Eun Sang Oh, Ju Hee Lee, Sung-Sik Han, Sang-Jae Park, Yang-Gun Suh, Sung Ho Moon, Sang Soo Kim, Dae Yong Kim

**Affiliations:** 1 Center for Liver Cancer, Research Institute and Hospital, National Cancer Center, Goyang, Republic of Korea; 2 Center for Proton Therapy, Research Institute and Hospital, National Cancer Center, Goyang, Republic of Korea

**Keywords:** pancreas cancer, proton beam therapy, overall survival

## Abstract

**Purpose:**

To evaluate the clinical effectiveness and feasibility of simultaneous integrated boost-proton beam therapy in patients with localized pancreatic cancer.

**Methods:**

Thirty-seven patients with localized pancreatic cancer underwent simultaneous integrated boost-proton beam therapy, and 8 (21.6%) patients received induction chemotherapy. The internal target volume was obtained by summing the gross tumor volumes in exhalation phase computed tomography images. Planning target volume 1 included internal target volume plus 3 to 5 mm margins, excluding the 5 mm expanded volume of gastrointestinal structures, and planning target volume 2 included the internal target volume plus 7 to 12 mm margins. The prescribed doses to planning target volume 1 and planning target volume 2 were 45 GyE (equivalent dose in 2 Gy, 54.4 GyE_10_) and 30 GyE (equivalent dose in 2 Gy, 32.5 GyE_10_) in 10 fractions, respectively.

**Results:**

Overall, treatment was well tolerated, with no grade of toxicity ≥3. Median overall survival was 19.3 months, and 1-year local progression-free survival, relapse-free survival, and overall survival rates were 64.8%, 33.2%, and 75.7%, respectively. Patients treated with simultaneous integrated boost-proton beam therapy after induction chemotherapy had a significantly higher median overall survival time compared to those with simultaneous integrated boost-proton beam therapy alone (21.6 months vs 16.7 months, *P* = .031). Multivariate analysis showed that induction chemotherapy was a significant factor for overall survival (*P* < .05).

**Conclusions:**

Simultaneous integrated boost-proton beam therapy could be feasible and promising for patients with localized pancreatic cancer.

## Introduction

At diagnosis, approximately 30% of patients with pancreatic cancer present with locally advanced disease.^1^ Although chemotherapy and/or radiotherapy (RT) have usually been performed, the role of RT is controversial because of conflicting results from clinical trials over the past decades comparing concurrent chemoradiotherapy (CRT) with chemotherapy alone in these patients.^2,3^ In the LAP07 trial,^3^ although CRT after induction chemotherapy did not show a survival benefit compared to chemotherapy alone, CRT resulted in a significantly longer period without treatment (6.1 months vs 3.7 months) and reduction in the local tumor progression (32% vs 46%; *P* < .05 for each). Additionally, several autopsy studies demonstrated that approximately 30% of patients with pancreatic cancer had no evidence of distant metastases at the time of death.^4,5^ One population-based study showed that 41% of patients with locally advanced cancer treated with chemotherapy died without evidence of distant metastases.^6^ These findings suggested that RT could be a valuable treatment option for selected patients with locally advanced disease.

When administering RT for patients with locally advanced disease, conventional fractionated courses of RT with concurrent chemotherapy have been typically used which has been associated with a significant grade 3 or 4 toxicity rate and a median overall survival (OS) of 9 to 15 months.^2,3,7,8^ With recent advances in RT techniques, intensity-modulated RT (IMRT), stereotactic body RT (SBRT), and proton beam therapy (PBT) can deliver high doses to the tumor as well as minimizing the radiation dose to surrounding normal tissues.^9–17^ Because of the apparent physical properties of proton beams that can deposit high doses of radiation to the target without an exit dose outside the target, PBT has been attracting attention. Conceptually, PBT has potential advantages of an accelerated RT, known as simultaneous integrated boost (SIB), which different doses can be delivered simultaneously to different targets. That is, higher doses can be delivered to the tumor, while lower doses can be delivered simultaneously to surrounding normal tissues, such as gastrointestinal structures close to the tumor. This accelerated hypofractionated RT can potentially improve the therapeutic ratio compared to conventional fractionated RT because it can reduce radiation damage to surrounding normal tissues, shorten overall treatment time, and avoid the need for prolonged chemotherapy breaks. Since June 2013, SIB-PBT has been applied for patients with localized pancreatic cancer in our institution, and the aim of this study was to evaluate the clinical effectiveness and safety of SIB-PBT in these patients.

## Materials and Methods

### Patients

Between June 2013 and July 2016, forty-one consecutive patients with pancreatic cancer were treated with SIB-PBT at our institute. Among them, there were 4 patients who had locoregional recurrent disease after surgical resection (n = 2), neuroendocrine carcinoma (n = 1), or distant metastasis (n = 1). The remaining 37 patients were retrospectively analyzed according to the guidelines of National Cancer Center (NCC) institutional review board (NCC20180158), and informed consent was not required because of the retrospective nature of this study.

All patients were given physical examinations, and complete blood count, liver function test, measurement of serum CA 19-9, chest radiography, computed tomography (CT) of the abdomen and pelvis, and/or positron-emission tomography (PET) were performed. All tumors were staged using the American Joint Committee on Cancer, Sixth edition, and were classified as stage cT4 (unresectable disease) based on the CT scans, with tumor extension to the celiac axis or superior mesenteric artery or occlusion of the superior mesenteric–portal venous confluence. Positive lymph node involvement was defined by the presence of a lymph node of at least 1 cm in the short axis, with a spiculated or indistinct border, or with a mottled heterogenic pattern on CT and/or PET scans (n = 35).^18^

### Treatment

**Figure 1.**
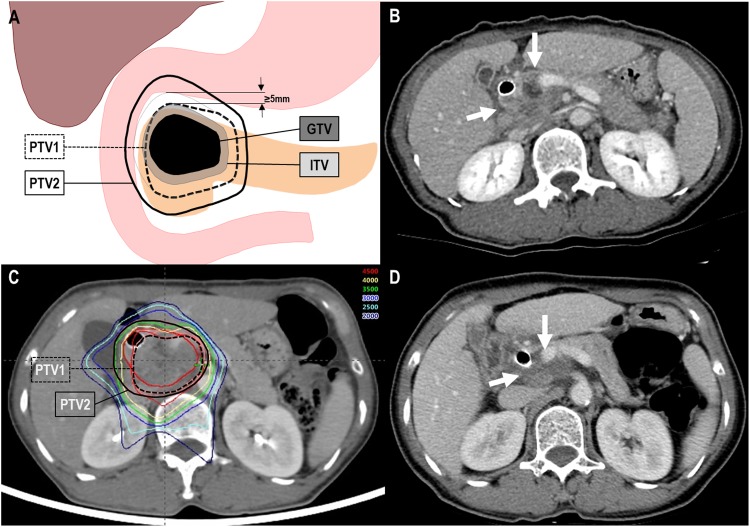
Partial response (PR) of a primary tumor to simultaneous integrated boost-proton beam therapy (SIB-PBT). (A) Definition of target volumes depending on the proximity of gastrointestinal structures, (B) pretreatment CT scans showing the primary tumor (arrow), (C) the patient underwent SIB-PBT, and (D) CT scans 3 months after SIB-PBT demonstrating PR of the primary tumor (arrow). CT indicates computed tomography; GTV, gross tumor volume; ITV, internal target volume; PTV, planning target volume.

For SIB-PBT planning, contrast-enhanced 4-dimensional CT images, with a 2.5-mm slice thickness, were obtained under shallow respiration while monitoring with a real-time position management (RPM) system (Varian Medical Systems, Palo Alto, California), and the CT images in 10 equally spaced respiratory phases were reconstructed. The average intensity projection (AIP) CT images were reconstructed using the CT images in gated (exhalation) phases (30% of the total respiratory cycle). The sum of contours of organs at risk (OARs) in each CT images during gated phase was delineated in AIP-CT image to account for residual organ motion. All tumors detected in AIP-CT images were defined as the gross tumor volume (GTV), and the sum of the GTVs in each CT images of the gated phases was defined as the internal target volume (ITV). Similar to other studies,^10–12^ clinical target volume expansion from GTV was not utilized. Planning target volume 1 (PTV1) included the ITV with a margin of 3 to 5 mm in all directions, excluding the 5 mm expanded volume of gastrointestinal structures to avoid gastrointestinal toxicity. The PTV2 included the ITV with a margin of 7 to 12 mm in all directions. The definition of target volumes is illustrated in Figure 1. Planning for SIB-PBT (Eclipse treatment planning system, version 8.1; Varian Medical System) was undertaken using 2 nonplanar or coplanar beams of 230 MeV protons (Proteus 235; Ion Beam Applications, S.A., Louvain-la-Neuve, Belgium) covering the PTV2 and 1 beam covering the PTV1, which was manually selected based on geometrical relationships of PTVs and OARs. The proximal, distal, border smoothing, smearing, and aperture margins of proton beams using the double scattering mode to the PTV were set to 5 to 10 mm each by considering the uncertainties by inter- and intrafractional organs motion. The doses for the target volumes and OARs were expressed in Gray equivalents (GyE = proton physical dose [in Gray] × relative biologic effectiveness [1.1]), and the equivalent dose in 2 Gy fractions (EQD2, GyE_10_ or GyE_3_) was calculated using a linear quadratic model with α–β ratios of 10 and 3 for acute and late effects on tumor and OARs, respectively, EQD2 = total dose [GyE, physical dose × 1.1] × (fraction dose [GyE] +10) / (2 + α/β).^19^ The beam weights of the plan were optimized to maximize the coverage of the target volumes and minimize the maximal doses of OARs, and the SIB-PBT plan was designed with the intent to cover at least 90% of the PTV1 and PTV2 with 100% of each prescribed dose and with minimum and maximum doses of >80% and <110%, respectively. The prescribed doses for PTV1 and PTV2 were 45 GyE (EQD2, 54.4 GyE_10_) and 30 GyE (EQD2, 32.5 GyE_10_) in 10 fractions, 5 times a week, respectively (Figure 1). The dose–volume constraints for the normal tissues have been described in our previous reports^9,20–23^: The maximum dose to the spinal cord were <27 GyE; the absolute volumes of the stomach and esophagus receiving ≥37 GyE were <2 cm^3^; the absolute volumes of the duodenum and bowel receiving ≥35 GyE were <2 cm^3^; the relative volumes of the liver receiving ≥27 GyE were below 60%; and the relative volumes of the kidney receiving ≥18 GyE were below 35%. The dose–volumetric parameters for target volumes and OARs are summarized in Supplementary Table 1. To reduce stomach movement and interfractional position problems for all patients, fasting was required at least 4 hours prior to treatment. At each treatment, digital orthogonal fluoroscopy was used to verify each patient’s position and the isocenter, and irradiation was delivered during the exhalation phase using the RPM system.

Of the 37 patients, 8 (21.6%) patients received a median of 4 cycles (range, 2-10) of induction chemotherapy, with 5-fluorouracil (5-FU), irinotecan and oxaliplatin (n = 4), gemcitabine and erlotinib (n = 3), and gemcitabine and cisplatin (n = 1) prior to SIB-PBT. Median interval from the date of the start of induction chemotherapy to SIB-PBT was 4.2 months (range, 1.8-7.1). During SIB-PBT, 31 (83.8%) patients received concurrent chemotherapy, with capecitabine (n = 29) and 5-FU (n = 2); the remaining 6 (16.2%) patients did not receive concurrent chemotherapy due to poor performance status because of advanced age (n = 5) and refusal (n = 1). After completion of SIB-PBT, patients who had resectable disease were considered for surgical resection, whereas patients who still had unresectable disease were considered for maintenance chemotherapy until disease progression or treatment-limiting toxicity. Chemotherapy regimens were chosen according to physician preference, and patients who refused further chemotherapy or had poor performance status received supportive care.

### Follow-Up and Statistical Considerations

The assessment of patients was performed weekly during SIB-PBT, 1 month after completion of SIB-PBT, every 2 to 3 months in the first 3 years, and every 6 months thereafter. Follow-up evaluations included a physical examination, complete blood count, liver function tests, measurement of serum CA 19-9, chest X-ray, and abdominopelvic CT scan. The responses of the primary tumor were defined as the maximal tumor response observed during the follow-up period in the absence of a progressive disease, which was assessed according to the Response Evaluation Criteria in Solid Tumours criteria^24^ by comparing CT scans before and after SIB-PBT. The objective response rates were the sum of the partial response (PR) and complete response (CR) rates. Patients who had PR or CR were defined as “responders,” and those who had stable disease (SD) or progressive disease (PD) were defined as “nonresponders.” Acute hematological and nonhematological toxicities occurring within 3 months of PBT in the absence of disease progression were assessed using the Common Terminology Criteria for Adverse Events (v4.0).

Recurrence was demonstrated by radiological findings, such as increased size with time and/or pathologic findings. Local failure was defined as progression of the primary tumor or recurrence at the primary tumor bed, regional failure was defined as progression or recurrence of disease in regional lymph nodes and soft tissues located near the primary tumor, while distant failure was defined as the development of distant metastasis. The OS, relapse-free survival (RFS), and locoregional progression-free survival (LPFS) were defined as the intervals from the date of start of induction chemotherapy or SIB-PBT (whichever came first) to the date of death or last follow-up, any detection of recurrence, and detection of locoregional progression, respectively. The probabilities of OS were calculated using the Kaplan-Meier method. Univariate analysis of factors related to OS were evaluated using log-rank tests, and multivariate analysis was performed using Cox proportional hazard model with a stepwise forward selection procedure including all variables of *P* < .1 in univariate analysis. All statistical tests were 2 sided and were performed using STATA software (version 14.0; StataCorp, College Station, Texas). A *P* value <.05 was considered statistically significant.

## Results

Patient characteristics are summarized in Table 1. Clinical T classification was cT3, medically inoperable due to a combination of advanced age (range, 72-87 years) and comorbidities (eg, heart disease, diabetes, and/or chronic renal insufficiency) in 5 (13.5%) patients and cT4 in 32 (86.5%) patients. After completion of SIB-PBT, overall and primary tumor response was as follows: PR in 8 (21.6%) and 14 (37.8%) patients, SD in 17 (45.9%) and 23 (62.2%) patients, and PD in 12 (32.4%) and 0 (0%) patients, respectively (Figure 1). Of the 5 patients with cT3 disease, 3 patients received concurrent chemotherapy with capecitabine, and none of the patients received induction and maintenance chemotherapy. Of the 32 patients with cT4 disease, 2 patients underwent surgical resection with resection margin negative after SIB-PBT (Table 1). After completion of SIB-PBT, 24 patients received maintenance chemotherapy, with gemcitabine (n = 9); gemcitabine and erlotinib (n = 9); tegafur/gemeracil/oteracil (n = 2); 5-FU, irinotecan, and oxaliplatin (n = 2); gemcitabine and nab-paclitaxel (n = 1); and capecitabine (n = 1). The remaining 13 patients did not receive maintenance chemotherapy because of patient refusal (n = 6), advanced age (n = 6), and poor performance status (n = 1; Table 1).

**Table 1.** Patient Characteristics.

Characteristic	Distribution, n (%)
Gender	
Male	20 (54.1)
Female	17 (45.9)
Age, years	
Median (range)	72 (52-92)
<70	17 (45.9)
≥70	20 (54.1)
ECOG PS	
0	31 (83.8)
1	6 (16.2)
Histology	
Adenocarcinoma	37 (100)
Tumor location	
Head	23 (62.2)
Body/tail	14 (37.8)
Tumor size,^a^ cm	
Median (range)	3.6 (2.0-7.3)
<4	23 (54.1)
≥4	14 (37.8)
T classification	
T3	5 (13.5)
T4	32 (86.5)
N classification	
N0	34 (91.9)
N1	3 (8.1)
Pretreatment CA 19-9 level, U/mL	
Median (range)	35.2 (2.0-1707)
≤37	20 (54.1)
>37	17 (45.9)
Induction chemotherapy^b^	
No	29 (78.4)
Yes	8 (21.6)
Pre-SIB-PBT CA 19-9 level, U/mL	
Median (range)	34.0 (2.0-1707)
≤37	21 (56.8)
>37	16 (43.2)
Concurrent chemotherapy^c^	
No	6 (16.2)
Yes	31 (83.8)
Post-SIB-PBT CA 19-9 level, U/mL	
Median (range)	24.3 (2.0-705)
≤37	21 (56.8)
>37	16 (43.2)
Post-SIB-PBT surgery^d^	
No	35 (94.6)
Yes	2 (5.4)
Maintenance chemotherapy^e^	
No	13 (35.1)
Yes	24 (64.9)

Abbreviations: ECOG PS, Eastern Cooperative Oncology Group performance status; CA 19-9, carbohydrate antigen 19-9; SIB-PBT, simultaneous integrated boost-proton beam therapy.

^a^ Maximum diameter of the primary tumor.

^b^ 5-Flurouracil (5-FU), irinotecan, plus oxaloplatin (FOLFINOX; n = 4), gemcitabine plus erlotinib (GT; n = 3), and gemcitabine plus cisplatin (GP; n = 1).

^c^ Capectabine (n = 29) and 5-FU (n = 2).

^d^ Pylorus-preserving pancreaticoduodenectomy (n = 1) and distal pancreatectomy (n = 1).

^e^ Gemcitabine (n = 9), GT (n = 9), tegafur/gemeracil/oteracil (TS-1; n = 2), FOLFINOX (n = 2), gemcitabine plus nab-paclitaxel (n = 1), and capecitabine (n = 1).

**Figure 2.**
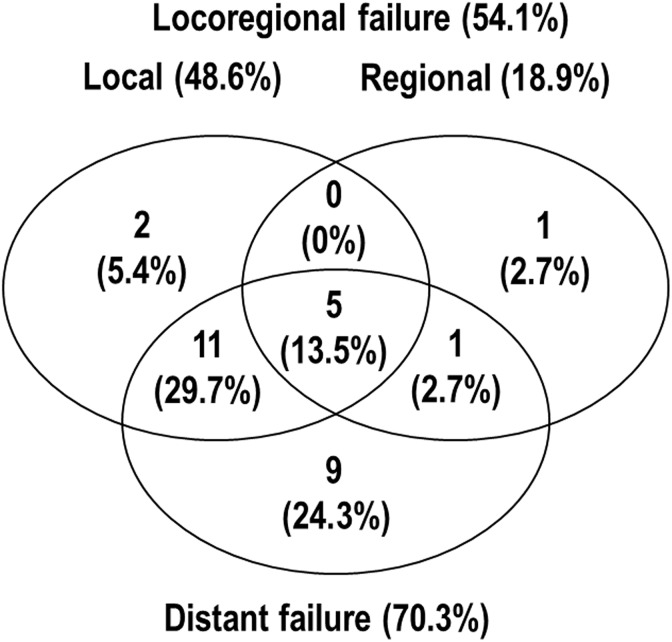
Patterns of treatment failure in all patients.

**Figure 3.**
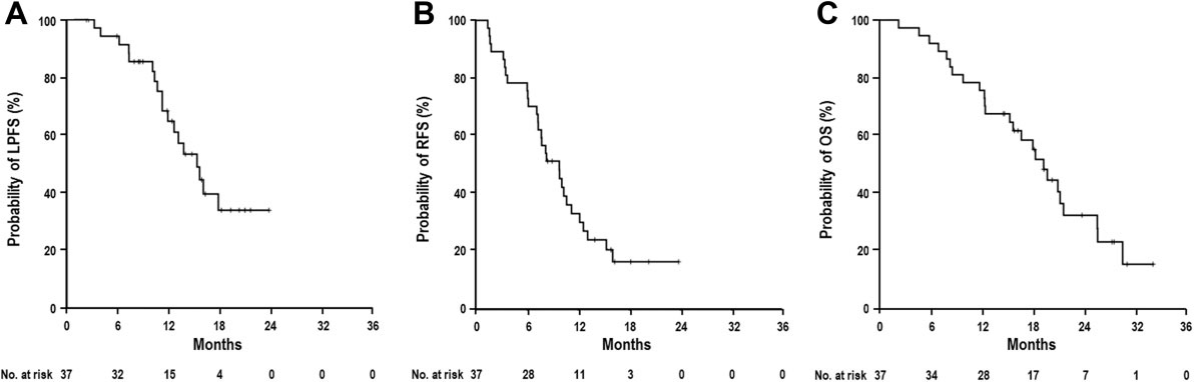
Locoregional progression-free survival (LPFS; A), relapse-free survival (RFS; B), and overall survival (OS; C) curves in all patients.

At the time of analysis, 25 patients had died from the disease and 12 remained alive. The median follow-up period was 16.7 months (range, 2.3-32.1 months) for all patients and 19.8 months (range, 14.5-32.1 months) for living patients. Of the 37 patients, 29 (75.6%) developed disease progression, including 18 (48.6%) with local progression, 7 (18.9%) with regional progression, and 26 (70.3%) with distant metastases (Figure 2). Two patients who received surgical resection remained alive at 18.2 months and 27.5 months after SIB-PBT, with regional recurrence at 10 months after surgical resection (n = 1) and controlled disease (n = 1), respectively. The median times of LPFS, RFS, and OS in all patients were 15.3 months (95% confidence interval [CI], 11.6-19.0 months), 9.8 months (95% CI, 7.1-12.4 months), and 19.3 months (95% CI, 16.5-22 months), respectively, and the actuarial 1-year LPFS, RFS, and OS rates were 64.8% (95% CI, 47.7%-81.9%), 33.2% (95% CI, 17.5%-48.9%), and 75.7% (95% CI, 61.8%-89.6%), respectively (Figure 3).

Univariate and multivariate analyses were performed to identify parameters predicting OS (Table 2). Univariate analysis demonstrated that induction chemotherapy, concurrent chemotherapy, and maintenance chemotherapy were significantly associated with OS (*P* < .05; Table 2). The patients who received surgical resection had a trend toward higher OS (remained alive at 18.2 and 27.5 months after SIB-PBT) than those who did not receive surgical resection (median, 18.3 months), but the difference was not significant due to the small number of patients who received surgical resection (n = 2; *P* = .139). Patient age (<70 years vs ≥ 70 years) showed marginal associations with OS (25.6 months vs 16.7 months, *P* = .066), whereas none of the other factors was significantly associated with OS (*P* > .05; Table 2). In multivariate analysis, induction chemotherapy was a significant factor independently associated with OS (*P* < .05; Table 2). Median OS time from SIB-PBT showed higher trend in the patients who received induction chemotherapy than those who did not (19 months vs 16.7 months), but the difference was not significant (*P* = 0.299).

**Table 2.** Univariate Analysis of Clinical Characteristics Associated With Overall Survival (OS).^a^

	Univariate		Multivariate	
Characteristic	OS, Median (95% CI), months	*P* Value^b^	Hazard Ratio (95% CI)	*P* Value^c^
Gender				
Male	20.9 (9.0-32.8)	.286	–	–
Female	18.3 (14.6-22.0)		–	
Age, years				
<70	25.6 (13.2-32.9)	.066	–	–
≥70	16.7 (12.0-21.3)		–	
ECOG PS				
0	19.3 (15.5-23.0)	.746	–	–
1	20.9 (-)		–	
Tumor location				
Head	18.0 (14.0-22.0)	.122	–	–
Body/tail	20.9 (18.1-23.7)		–	
Tumor size,^b^ cm				
<4	18.0 (14.2-21.8)	.259	–	–
≥4	20.9 (17.7-24.1)		–	
T classification				
T3	15.7 (4.2-27.2)	.156	–	–
T4	19.7 (16.4-23.0)		–	
N classification				
N0	19.3 (15.8-22.8)	.390	–	–
N1	25.6 (4.4-46.8)		–	
Pretreatment CA 19-9 level, U/mL				
≤37	16.7 (8.3-25.0)	.749	–	–
>37	19.7 (14.8-24.4)		–	
Induction chemotherapy				
No	16.7 (13.5-19.8)	.031	1.000	.040
Yes	21.6 (-)		0.317 (0.106-0.949)	
Pre-SIB-PBT CA 19-9 level, U/mL				
≤37	19.3 (12.6-25.9)	.719	–	–
>37	19.7 (15.6-23.8)		–	
Concurrent chemotherapy				
No	8.3 (2.9-13.7)	.036	–	–
Yes	20.9 (16.8-25.1)		–	
Post-SIB-PBT CA 19-9 level, U/mL				
≤37	19.3 (14.3-24.3)	.541	–	–
>37	21.6 (15.8-27.4)		–	
Primary tumor response				
Responder	21.2 (19.3-23.1)	.432	–	–
Nonresponder	16.7 (12.6-20.7)		–	
Post-SIB-PBT surgery				
No	18.3 (12.2-21.6)	.139	–	–
Yes	NR (-)		–	
Maintenance chemotherapy				
No	15.7 (8.2-23.2)	.044	–	–
Yes	21.2 (17.4-25.0)		–	

Abbreviations:CI, confidence interval; ECOG PS, Eastern Cooperative Oncology Group performance status; CA 19-9, carbohydrate antigen 19-9; NR, not reached; SIB-PBT, simultaneous integrated boost-proton beam therapy.

^a^ Responder denotes complete or partial response and nonresponder denotes stable disease or progressive disease.

^b^ Log-rank test.

^c^ Cox proportional hazards model.

During SIB-PBT, acute toxicities were transient, easily manageable, and caused no interruption in the treatment course, and the details of the distribution of acute toxicities are summarized in Table 3. The most common toxicities were grade 1 anemia (32.4%), grade 1 leukopenia (21.6%), and grade 1 abdominal pain (16.2%), and no cases of grade ≥3 acute toxicity were detected. No late radiation toxicities of grade ≥3, such as gastrointestinal bleeding or duodenal ulcer, were observed.

**Table 3.** Acute toxicities During Simultaneous Integrated Boost-Proton Beam Therapy.^a^

Type of Toxic Effect^b^	Grade 0, n (%)	Grade 1, n (%)	Grade 2, n (%)	Grade 3, n (%)	Grade 4, n (%)	Grade 5, n (%)
Hematologic toxicity						
Leukopenia	28 (75.7)	8 (21.6)	1 (2.7)	0 (0)	0 (0)	0 (0)
Anemia	22 (59.4)	12 (32.4)	3 (8.1)	0 (0)	0 (0)	0 (0)
Thrombocytopenia	36 (97,3)	1 (2.7)	0 (0)	0 (0)	0 (0)	0 (0)
Nonhematologic toxicity						
Hand-foot syndrome	37 (100)	0 (0)	0 (0)	0 (0)	0 (0)	0 (0)
Anorexia	30 (81.1)	4 (10.8)	3 (8.1)	0 (0)	0 (0)	0 (0)
Vomiting	32 (86.5)	3 (8.1)	2 (5.4)	0 (0)	0 (0)	0 (0)
Diarrhoea	37 (100)	0 (0)	0 (0)	0 (0)	0 (0)	0 (0)
Abdominal pain	31 (83.8)	6 (16.2)	0 (0)	0 (0)	0 (0)	0 (0)
Stomatitis	35 (94.6)	1 (2.7)	1 (2.7)	0 (0)	0 (0)	0 (0)

^a^ Some patients experienced more than 1 toxicity.

^b^ National Cancer Institute Common Terminology Criteria for Adverse Events, version 3.0.

## Discussion

In patients with locally advanced pancreatic cancer, the role of RT has been disputed because of conflicting results with regard to OS benefit, whether CRT was added or chemotherapy alone. In addition, there was the concern of significant high toxicity following CRT.^2,3,7,8^ In particular, the Eastern Cooperative Oncology Group 4201 trial demonstrated a 77% incidence of grade ≥3 toxicity in patients with locally advanced disease receiving RT with concurrent gemcitabine. These previous trials used conventionally fractionated RT with large RT volume, including elective nodal irradiation.^2,3,7^ Recently, conventional or hypofractionated IMRT with 5 to 39 fractions or SBRT with 1 to 8 fractions, using a limited RT volume without elective nodal irradiation, has been used to improve the sparing of surrounding normal structures while increasing the dose to the target volume. This treatment has resulted in median OS of 6 to 20 months and grade ≥3 toxicity of 0% to 26%.^9–15,25^ Several studies have showed a dosimetric superiority of PBT compared to RT with X-ray, including IMRT, for the delivery of radiation dose to the target while significantly reducing exposure to surrounding normal tissues and the dosimetric feasibility of hypofractionated PBT for pancreatic cancer.^26,27^ Terashima *et al* ^14^ reported on the outcomes for 50 patients with pancreatic cancer with locally advanced disease treated with PBT, with 50 to 70.2 GyE in 25 to 26 fractions, and concurrent gemcitabine. They found promising outcomes in terms of 1-year LPFS, RFS, and OS rates of 81.7%, 64.3%, and 76.8%, respectively, and grade ≥3 toxicity of 10%. In addition, Nichols *et al* ^15^ analyzed 22 patients with pancreatic and ampullary cancer who were treated with PBT, 50 to 59.4 GyE in 28 to 33 fractions, and concurrent capecitabine. They reported a favorable toxicity profile including no grade ≥3 gastrointestinal toxicity. In the present study, we applied SIB-PBT, prescribed 45 or 30 GyE in 10 fractions to target volumes depending on the closeness of the gastrointestinal structures, and observed a median OS of 19.7 months for patients with locally advanced disease (ie, cT4) and no grade ≥3 toxicity. Direct comparison of data among previous studies and those of our present study is difficult due to heterogeneous baseline characteristics, particularly in performance status and tumor burden (ie, tumor size, lymph node involvement) and the various agents and sequence of chemotherapy administered. However, the median OS and incidence of grade ≥3 toxicity in the present study were at the higher and lower end of the wide range reported previously, respectively.^9–15,25^

Due to the high propensity of patients with locally advanced disease to develop distant metastases, induction chemotherapy before CRT has been proposed to select the subset of patients who will benefit from RT.^28–30^ Although several retrospective studies^28,29^ showed an OS benefit of CRT after induction chemotherapy compared to chemotherapy alone, the recent randomized LAP07 trial^3^ did not show an additional benefit by adding CRT after induction chemotherapy. However, the LAP07 trial demonstrated a significant decrease in the rate of local progression using CRT after induction chemotherapy compared to chemotherapy alone, and recent cohort studies showed an OS benefit of CRT after induction chemotherapy compared to chemotherapy alone.^31,32^ In the present study, the patients treated with SIB-PBT after induction chemotherapy had a significantly higher median OS time from commencement of treatment and higher trend in median OS time from SIB-PBT compared to those treated with SIB-PBT alone (21.6 months vs 16.7 months, *P* = .031, and 19 months vs 16.7 months, *P* = .299, respectively; Table 2). Although the study population was small and did not include those patients who were treated with chemotherapy alone, these findings implied that induction chemotherapy may help to select the patients who benefit from RT.

Chemotherapy and/or RT are often the treatment of choice for patients with medically inoperable disease, and its outcomes have been reported. Low-dose gemcitabine may improve survival compared to best supportive care in unresectable disease (median OS, 7.6 months vs 2.3 months, *P* < .05),^33^ and chemotherapy and/or RT has shown a median OS time of 8.6 to 12.2 months in medically inoperable disease.^34,35^ Recently, SBRT has been tried in medically inoperable disease based on its promising outcomes, in terms of high local control with minimal toxicity; it was used in unresectable disease, and it showed a median OS time of 6.4 to 7.6 months.^36,37^ To date, the question remains of how best to manage these patients who cannot tolerate aggressive treatments, such as surgery, chemotherapy, and/or a conventional course of RT. In the present study, SIB-PBT showed a median OS time of 15.7 months in medially inoperable disease. Although the number of these patients was small (n = 5), outcomes of SIB-PBT for patients with medically inoperable disease were promising.

This study was retrospective and thus had certain inherent limitations. First, our data were from a single institutional study with a relatively small and heterogeneous population, which included patients with medically inoperable and locally advanced disease and had the heterogeneity of various chemotherapeutic agents and sequence; thus, the effects of systemic chemotherapy and probable selection bias were not thoroughly evaluated. Second, the assessment of toxicity in a retrospective analysis inherently underestimates risks owing to incomplete reporting of side effects in clinic notes, recall bias, and lack of continuity of follow-up at 1 institution to capture all possible adverse events. Despite these study limitations, SIB-PBT with 10 factions offers the advantage of delivering RT over 2 weeks and thereby minimizes the delay in administration of chemotherapy. In addition, due to superior dose localization of the proton beams to the target than is achieved by X-ray, SIB-PBT can potentially result in minimizing RT dose to surrounding gastrointestinal structures. In the present study, the median OS in the patients with localized inoperable disease treated with SIB-PBT was 19.3 months, and grade ≥3 toxicity was not observed. However, because of the aforementioned limitations and lack of comparison of RT with X-ray, such as IMRT and SBRT, further large-scale prospective studies are warranted, including combinations of SIB-PBT and modern systemic chemotherapy regimens, such as 5-FU, irinotecan plus oxaliplatin, and gemcitabine plus nab-paclitaxel,^38,39^ and/or dose escalation using SIB-PBT techniques.

In conclusion, we found that SIB-PBT for patients with localized pancreatic cancer showed promising results, including a median OS time of 19.3 months and no grade ≥3 toxicity. Because we could not analyze subgroups in detail due to the relatively small number of the study population and heterogeneity of pre- and post-RT treatments, further larger scaled prospective studies are warranted. However, our data suggest that SIB-PBT could be a feasible and promising component of combination therapy for these patients.

## Supplementary Material

Supplementary material

## Abbreviations

AIP: average intensity projection
CI: confidence interval
CR: complete response
CRT: concurrent chemoradiotherapy
CT: computed tomography
CTCAE: common terminology criteria for adverse events
GTV: gross tumor volume
GyE: Gray equivalent
IMRT: intensity-modulated radiotherapy
ITV: internal target volume
LPFS: locoregional progression-free survival
OARs: organs at risk
OS: overall survival
PBT: proton beam therapy
PD: progressive disease
PET: positron-emission tomography
PR: partial response
PTV: planning target volume
RFS: relapse-free survival
RPM: real-time position management
RT: radiotherapy
SBRT: stereotactic body RT
SD: stable disease
SIB: simultaneous integrated boost.
