# Supplementary material for: Effectiveness and Safety of Simultaneous Integrated Boost-Proton Beam Therapy for Localized Pancreatic Cancer
Source: Technol Cancer Res Treat. 2018 Jul 1;17:1533033818783879. doi: 10.1177/1533033818783879 (PMC6048612; doi:10.1177/1533033818783879)
Supplement: Supplementary material [file Supplementary_Table_1.pdf]

Supplementary Table 1. A summary of dose-volumetric parameters.

Variable		Median (range)
PTV1	D95 (%)	96.9 (89.6 – 100.4)
	D90 (%)	98.6 (92.8 – 113.4)
	Dmin (%)	80.9 (70.6 – 91.8)
	Dmax (%)	103.6 (101.9 – 113.4)
PTV2	D95 (%)	102.7 (100.8 – 105.9.)
	D90 (%)	105.1 (101.3 – 111.4)
	Dmin (%)	97.8 (94.1 – 99.8)
	Dmax (%)	155.4 (152.9 – 170.1)
Liver	Dmean (GyE)	2.3 (0.76 – 7.5)
	V27 (%)	0.6 (0 – 5.4)
Stomach	D2cm^3 (GyE)	32.3 (27.1 – 35.0)
Duodenum	D2cm^3 (GyE)	33.6 (32.7 – 34.4)
Bowel	D2cm^3 (GyE)	32.4 (30.4 – 33.6)
Esophagus	D2cm^3 (GyE)	4.1 (0.2– 32.5)
Kidney, Right	Dmean (GyE)	7.6 (4.1– 12.1)
	V18 (%)	0 (0– 11.7)
Kidney, Left	Dmean (GyE)	8.4 (0.9– 12.8)
	V18 (%)	0 (0– 38.1)
Spinal Cord	D2cm^3 (GyE)	5.9 (3.7– 13.0)

Abbreviations: PTV, planning target volume; D95 and D90, minimal target dose received by at least 95% and 90% of the PTV, respectively; Dmin, Dmax, and Dmean, minimum, maximum, and mean dose to PTV and the organs at risk, respectively; V18 and V27, percentage of irradiated volume receiving ≥18 and ≥27 GyE, respectively;; and D2cm^3, the delivered dose to the organs at risk of 2 cm^3 volume, respectively.
